# High performance Ce-doped ZnO nanorods for sunlight-driven photocatalysis

**DOI:** 10.3762/bjnano.7.125

**Published:** 2016-09-26

**Authors:** Bilel Chouchene, Tahar Ben Chaabane, Lavinia Balan, Emilien Girot, Kevin Mozet, Ghouti Medjahdi, Raphaël Schneider

**Affiliations:** 1 Unité de Recherche Synthèse et Structure de Nanomatériaux UR 11 ES 30. Université de Carthage, Faculté des Sciences de Bizerte, 7021 Jarzouna, Bizerte, Tunisia; 2 Institut de Science des Matériaux de Mulhouse (IS2M), CNRS, UMR 7361, 15 rue Jean Starcky, 68093 Mulhouse, France; 3 Université de Lorraine, Laboratoire Réactions et Génie des Procédés (LRGP), UMR 7274, CNRS, 1 rue Grandville, BP 20451, 54001 Nancy Cedex, France; 4 Institut Jean Lamour (IJL), Université de Lorraine, CNRS, UMR 7198, CNRS, BP 70239, 54506 Vandoeuvre-lès-Nancy Cedex, France

**Keywords:** Ce doping, photocatalysis, solvothermal synthesis, ZnO rods

## Abstract

Ce-doped ZnO (ZnO:Ce) nanorods have been prepared through a solvothermal method and the effects of Ce-doping on the structural, optical and electronic properties of ZnO rods were studied. ZnO:Ce rods were characterized by XRD, SEM, TEM, XPS, BET, DRS and Raman spectroscopy. 5% Ce-doped ZnO rods with an average length of 130 nm and a diameter of 23 nm exhibit the highest photocatalytic activity for the degradation of the Orange II dye under solar light irradiation. The high photocatalytic activity is ascribed to the substantially enhanced light absorption in the visible region, to the high surface area of ZnO:Ce rods and to the effective electron–hole pair separation originating from Ce doping. The influence of various experimental parameters like the pH, the presence of salts and of organic compounds was investigated and no marked detrimental effect on the photocatalytic activity was observed. Finally, recyclability experiments demonstrate that ZnO:Ce rods are a stable solar-light photocatalyst.

## Introduction

Due to the increasing pollution of water and air, there is a growing interest to find sustainable, efficient and cheap solutions to purify these media. In this context, semiconductor photocatalysis technology has been thoroughly studied since 1972 to solve environmental and energy challenges [1]. After absorption of light with an adequate wavelength by the semiconductor, electrons (e^−^) are promoted from the valence band (VB) to the conduction band (CB) and holes (h^+^) generated in the VB to form electron–hole pairs. Most of these e^−^/h^+^ pairs recombine and only a small percentage migrate to the surface of the photocatalyst where they can be trapped by water and oxygen to generate hydroxyl ^•^OH and superoxide O_2_^•−^ radicals, respectively. These highly oxidizing species are responsible of the conversion of organic compounds into carbon dioxide, water, and inorganic salts.

Zinc oxide (ZnO) is one of the most widely investigated semiconductor photocatalyst owing to its availability, weak toxicity, stability and relatively good resistance to photocorrosion [2–7]. However, to efficiently use ZnO in practice as an air and water decontamination agent, two drawbacks should be overcome. First, ZnO is a wide bandgap material (*E*_g_ = 3.37 eV at room temperature) and can only be activated by UV light with a wavelength equal or lower than 385 nm to trigger the e^−^/h^+^ separation. Second, ZnO suffers from a low photocatalytic efficiency due to the easy recombination of the photogenerated e^−^/h^+^ pairs, which limits the diffusion of charge carriers from the bulk to the surface of ZnO.

To shift ZnO absorption to the visible part of the solar spectrum and decrease charge recombination on the surface of the photocatalyst, doping of ZnO has been widely investigated in recent years [8–13]. Doping means introducing metal (Cu, Mn, Co, Al, …) or non-metal elements (C, N, S,…) into the ZnO crystal lattice to change its properties. Doping of ZnO will increase the material defects and thus decrease e^−^/h^+^ recombinations but also displace its adsorption to the visible range. In recent years, rare earth doping of ZnO has attracted significant attention, especially with cerium. Ce^3+^ possesses shielded 4f levels, which allow various well-defined narrow optical transitions between the spin-orbit levels and thus split the bandgap of ZnO into sub-gaps. For this reason, Ce^3+^ is generally doped or associated to ZnO to improve the luminescence efficiency by energy transfer processes and this topic is becoming an exciting area of research for developing electronic and optical applications like sensors, light-emitting phosphors or flat panel displays [14–27]. Due to the defects induced in the ZnO crystalline structure by Ce doping and the ability of this element to trap photogenerated charge carriers, Ce-doped ZnO (ZnO:Ce) particles have also gained high interest for photocatalysis. Recently, a few Ce^3+^- or Ce^4+^-doped ZnO photocatalyst containing large particles of spherical or needle morphology have been developed and their ability to degrade cyanide anions [28] or organic dyes [29–33] like methylene blue or methyl-orange has been demonstrated. The preparation of Ce–Cu, Ce–Pd or Ce–Ag co-doped photocatalysts to enhance the solar or the visible light catalytic response was also reported [34–36]. The synthesis of particles with well-defined properties is also of high importance to control the photocatalytic activity. Their optical, chemical and electronic properties are actually strongly dependent on shape, size, crystalline structure, defect concentration, and surface area. Among the various ZnO nanostructures developed for photocatalytic applications, ZnO rods have attracted a high interest due to their stability, their large specific surface area that favor mass transfer and generate more reactive sites, and their weak sensibility to photocorrosion [37–48].

In this manuscript, a new solvothermal method for the preparation of small-sized ZnO:Ce rods with average length and diameter of ca. 130 nm and 23 nm, respectively, is first described. The effects of Ce-doping on ZnO rods size and morphology and on their optical, electronic and photocatalytic properties have been investigated. Our results demonstrate that Ce-doping improves the optical absorption ability toward visible light wavelengths and thus the photocatalytic performances. ZnO:Ce rods allow the complete degradation of the Orange II dye in ca. 80 min under solar light irradiation. The possible mechanism of photodegradation is discussed. Finally, ZnO:Ce rods are highly stable, so that they can be reused up to five times without significant performance loss, which is a very attractive feature for practical photocatalytic applications.

## Results and Discussion

### Structural and optical characterizations of Ce-doped ZnO rods

**Scheme 1:**
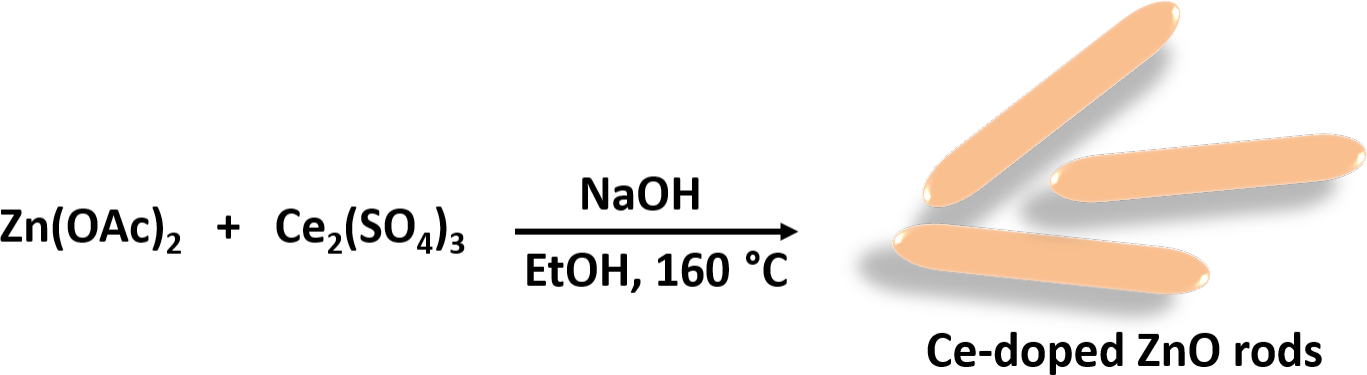
Schematic illustration of the synthesis of the Ce-doped ZnO rods.

Ce-doped ZnO rods have been synthesized by a solvothermal method in a Teflon-lined stainless steel autoclave at 160 °C for 24 h using Zn(OAc)_2_ and Ce_2_(SO_4_)_3_ as starting reagents (Scheme 1).

**Figure 1:**
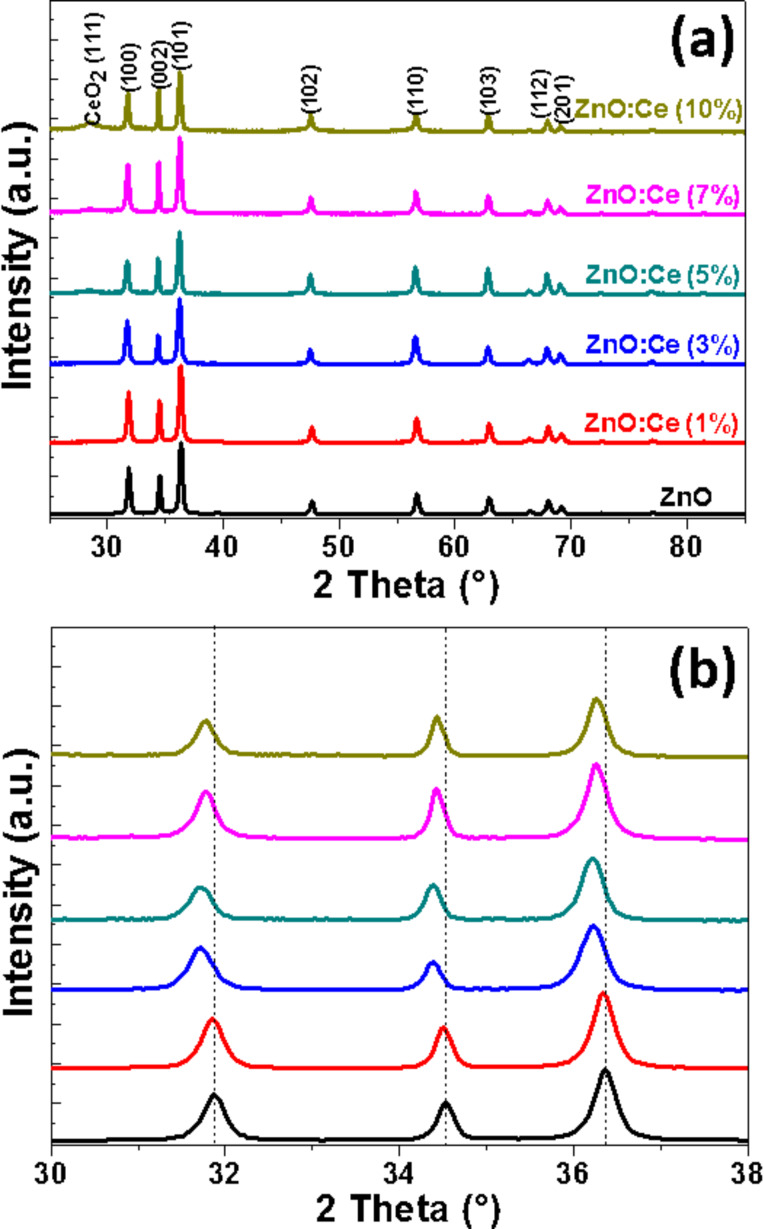
(a) XRD patterns of ZnO and Ce-doped ZnO rods and (b) magnification from 30 to 38°.

Figure 1a shows the X-ray diffraction (XRD) patterns of ZnO rods when varying the Ce-dopant percentage from 0 to 10. The XRD patterns show sharp peaks, indicating a high degree of crystallinity. The peaks located at 2θ values of 31.7, 34.3, 36.1, 47.5, 56.5, 62.7, 67.8 and 69° are ascribed to the (100), (002), (101), (102), (110), (103), (112) and (201) crystal planes of wurtzite ZnO (JCPDS No 36-1451). It should also be mentioned that for the highest degrees of doping (5, 7 and 10% in Ce doping), an additional and weak signal corresponding to the (111) diffraction plane of cubic CeO_2_ could be observed at 2θ = 28.3° (JCPDS No 34-0394), thus indicating the partial oxidation of Ce^3+^ into Ce^4+^ during the synthesis and the formation of CeO_2_. To demonstrate the incorporation of Ce^3+^ or Ce^4+^ in Zn^2+^ sites or interstitial sites in the ZnO lattice, the angle shift of the (100), (002) and (101) peaks as a function of doping percentage has been studied. As can be seen from Figure 1b, the position of these peaks is shifted toward lower angles when increasing the Ce doping, thus indicating its incorporation, at least partly, in the ZnO crystal lattice [17]. A slight increase of ZnO a and c lattice parameters is also observed as a result of the Ce doping because the radii of Ce^3+^ (0.103 nm) and Ce^4+^ (0.092 nm) are larger than that of Zn^2+^ (0.074 nm) (Table S1 in the Supporting Information File 1). This result is in good accordance with previous reports from the literature [14,34].

**Figure 2:**
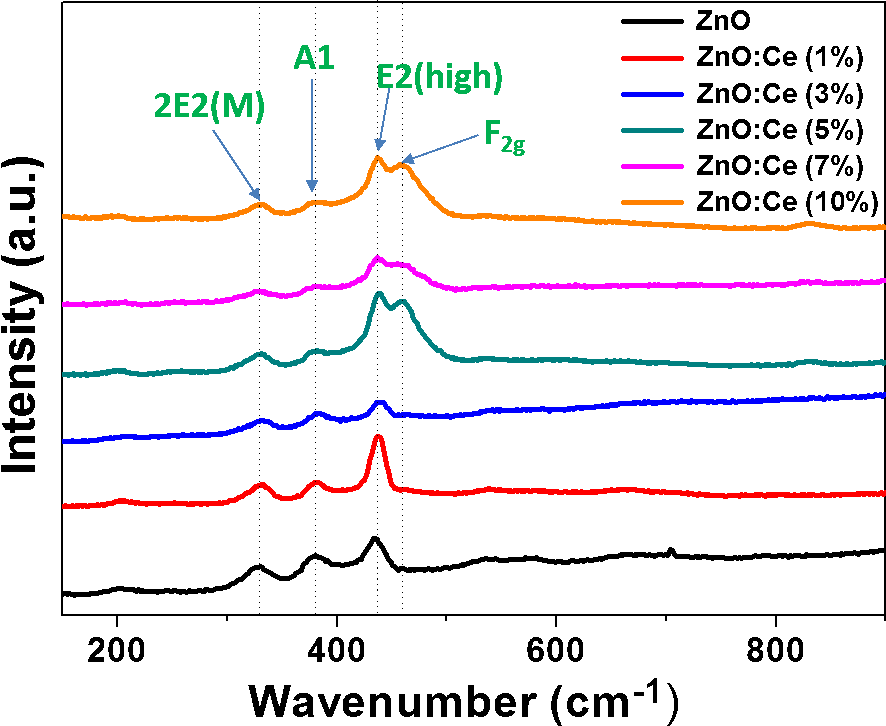
Raman spectra of ZnO and ZnO:Ce rods.

The partial oxidation of Ce^3+^ during the solvothermal synthesis and the formation of CeO_2_ was further confirmed by Raman spectroscopy and X-ray photoelectron spectroscopy (XPS) analysis. Figure 2 shows the Raman spectra of undoped and Ce-doped ZnO rods excited by the 532 nm line of a YAG laser. The peaks located at 332, 379, and 437 cm^−1^ can be assigned to 2*E*2, *A*1(*To*), and *E*2(*high*) vibration modes of wurtzite ZnO with *P*6_3_*mc* symmetry [49]. In the spectra of ZnO:Ce (5, 7 and 10% doping) materials, the signal observed at 457 cm^−1^ originates from the Raman active mode characteristic of CeO_2_ fluorite-structured materials with *F*2*g* symmetry and corresponds to the ceria Ce-O8 vibrational unit [50–51].

**Figure 3:**
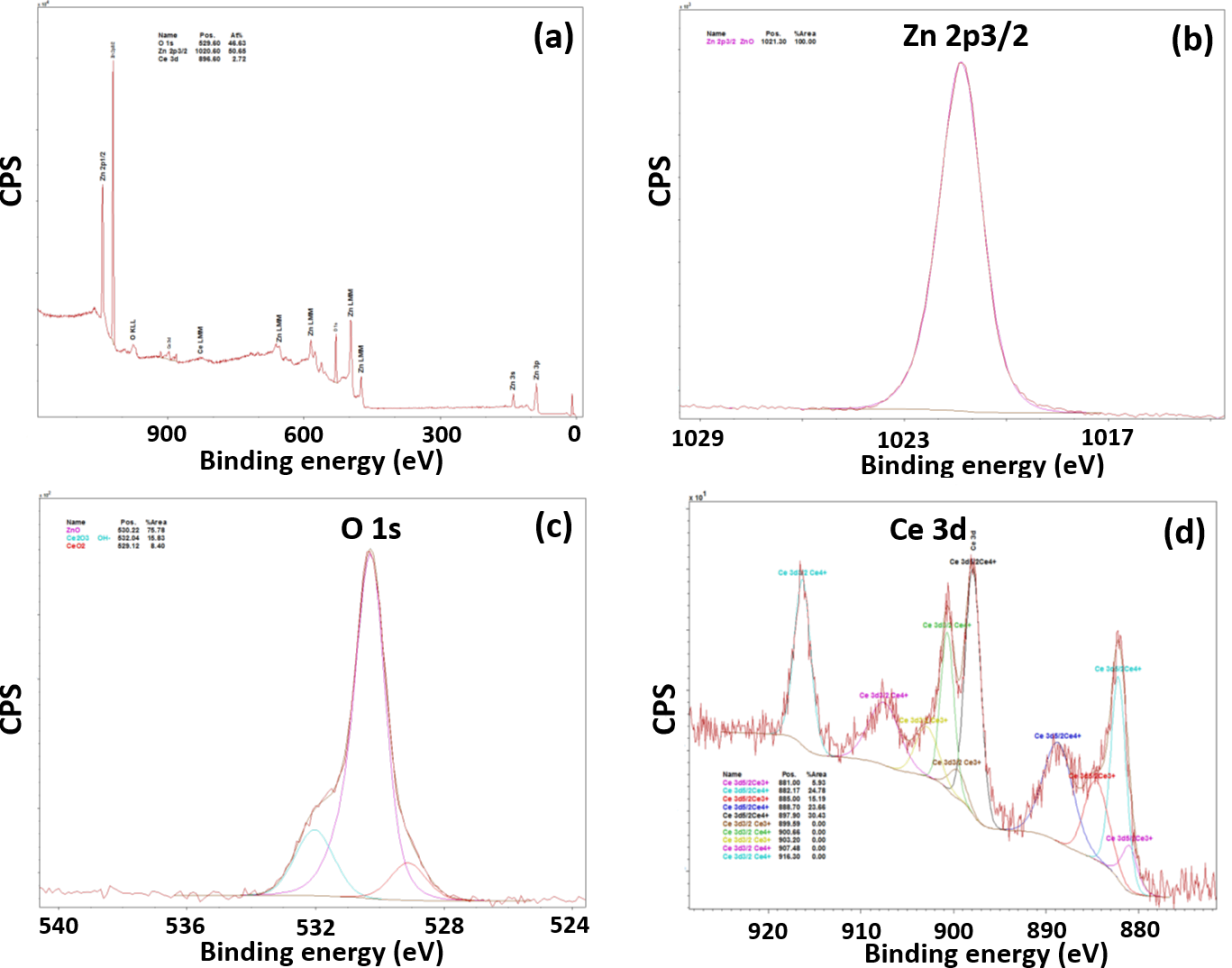
XPS analysis of the prepared ZnO:Ce (5%) rods. (a) Survey scan, and high resolution scans of (b) Zn 2p region, (c) O 1s region, and (d) Ce 3d region.

Further evidence of the presence of Ce^3+^ and Ce^4+^ in the materials produced was obtained by XPS analysis. Only the peaks of Zn 2p, O 1s and Ce 3d can be observed on the survey spectrum of the sample doped with 5% Ce (Figure 3a). The Zn 2p_3/2_ peak located at 1021.3 eV is characteristic of Zn–O bonds in the ZnO lattice (Figure 3b). The O 1s signal could be deconvoluted into three peaks (Figure 3c). The main signal located at 530.2 eV corresponds to O bound to Zn^2+^. The low and high energy components located at 529.1 and 532.0 eV can be assigned to Ce^4+^ and Ce^3+^ linked to O, respectively. Due to the co-existence of Ce^3+^ and Ce^4+^ in ZnO:Ce rods, the Ce 3d spectrum is complex and shows five doublets originating from the 3d_5/2_ and 3d_3/2_ spin–orbit split components (Figure 3d) [52–53]. The energy separations between the two spin–orbit levels were found to be of ca. 18.2 eV. The binding energy peaks at 881.0, 885.0, 899.6, and 903.2 eV correspond to Ce^3+^ species while the peaks at 882.1, 888.7, 897.9, 900.6, 907.5 and 916.3 eV are related to Ce^4+^ species. It is worth noting that the well separated peak at 916.3 eV is characteristic for the presence of Ce(+4) in the ZnO:Ce rods [53]. Finally, the relative contributions of Ce^4+^ and Ce^3+^ states at the surface of ZnO rods were estimated to be 79 and 21%, respectively.

**Figure 4:**
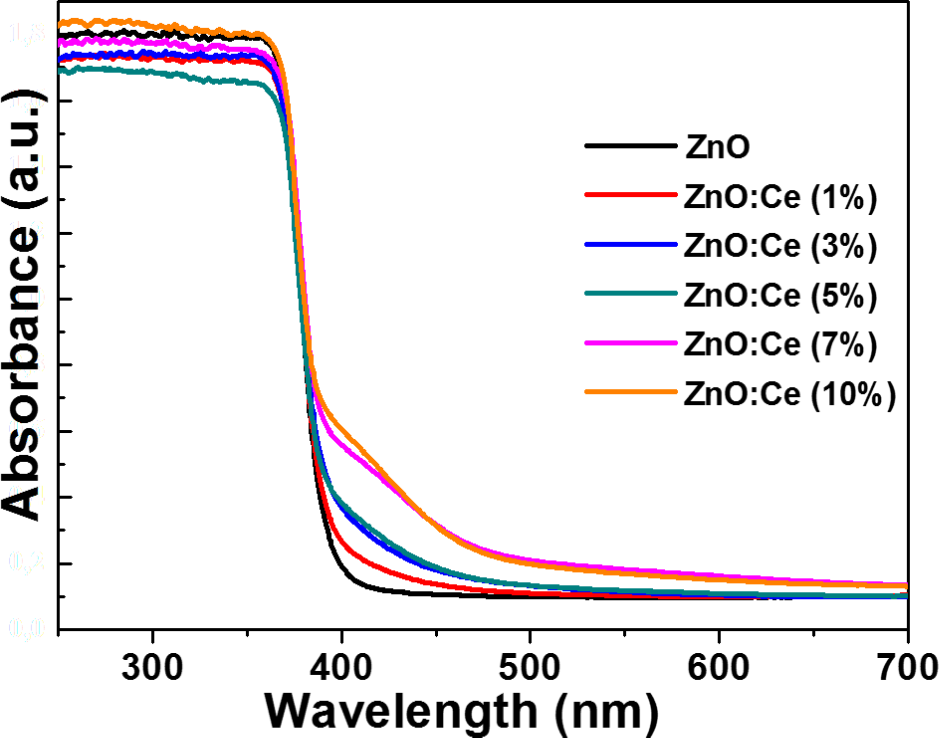
Room temperature UV–visible diffuse reflectance spectra of ZnO and Ce-doped ZnO rods.

To evaluate the photo-absorption behaviors of the ZnO:Ce materials, the UV–visible diffuse reflectance spectra (DRS) were recorded and shown in Figure 4 (undoped ZnO rods were used as reference). The absorption peak at ca. 370 nm for ZnO rods is assigned to the ground excitonic state of ZnO. By comparing the absorptions of ZnO and Ce-doped ZnO particles, it can be seen that the absorption intensity of ZnO:Ce rods in the range of 400–500 nm is increased with increasing Ce concentrations. The redshift in absorption probably originates from sub-bandgap transitions originating from Ce doping [34,54]. These electronic transitions from the VB of ZnO to the Ce energy levels require less energy than that of the VB to the CB of ZnO. Reflectance spectra have been converted to the absorbance spectra using the Kubelka–Munk function *F*_KM_(*R*) = (1 − *R*)^2^/2*R*, where *R* is the reflectance recorded for the sample (Figure S1 in the Supporting Information File 1). The bandgap energies determined by an extrapolation method vary between 3.29 and 3.30 eV for ZnO and ZnO:Ce rods and indicate that there are quite no changes in the ZnO bandgap when varying the dopant percentage in Ce, which is in good accordance with absorption results previously described and with a recent report from the literature [34].

**Figure 5:**
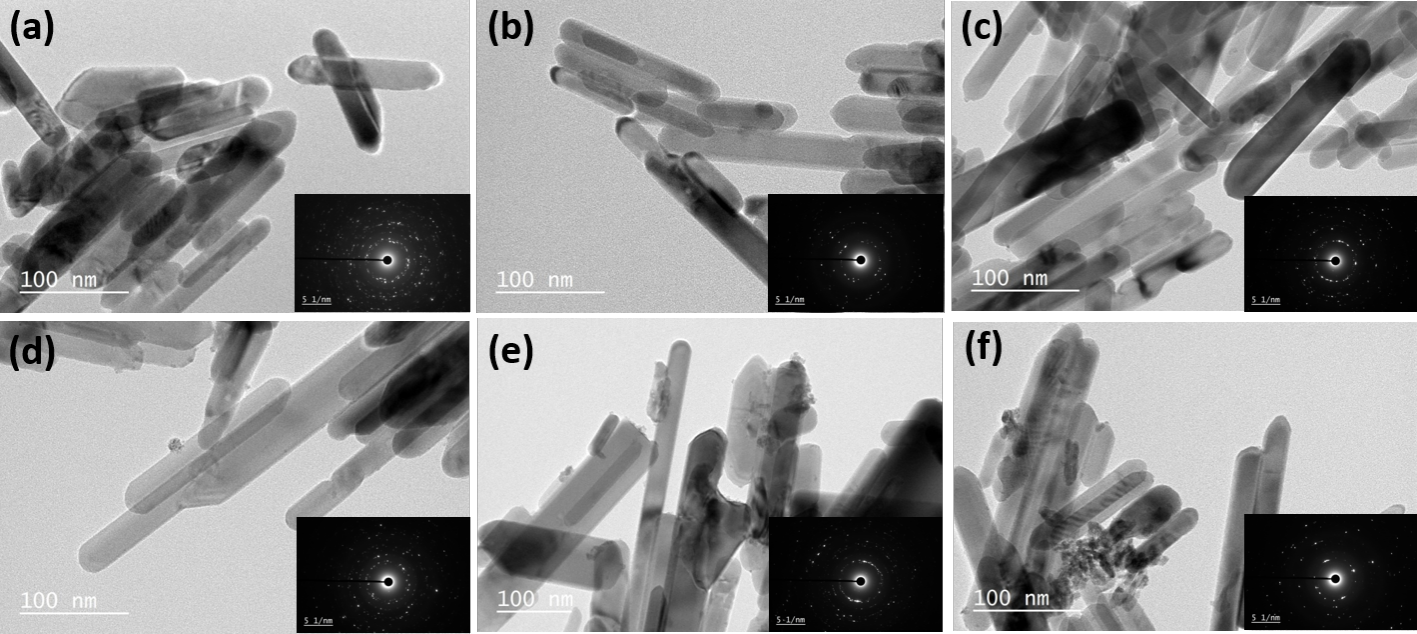
TEM images of (a) ZnO rods and (b–f) ZnO rods doped with 1, 3, 5, 7 and 10% Ce, respectively.

**Figure 6:**
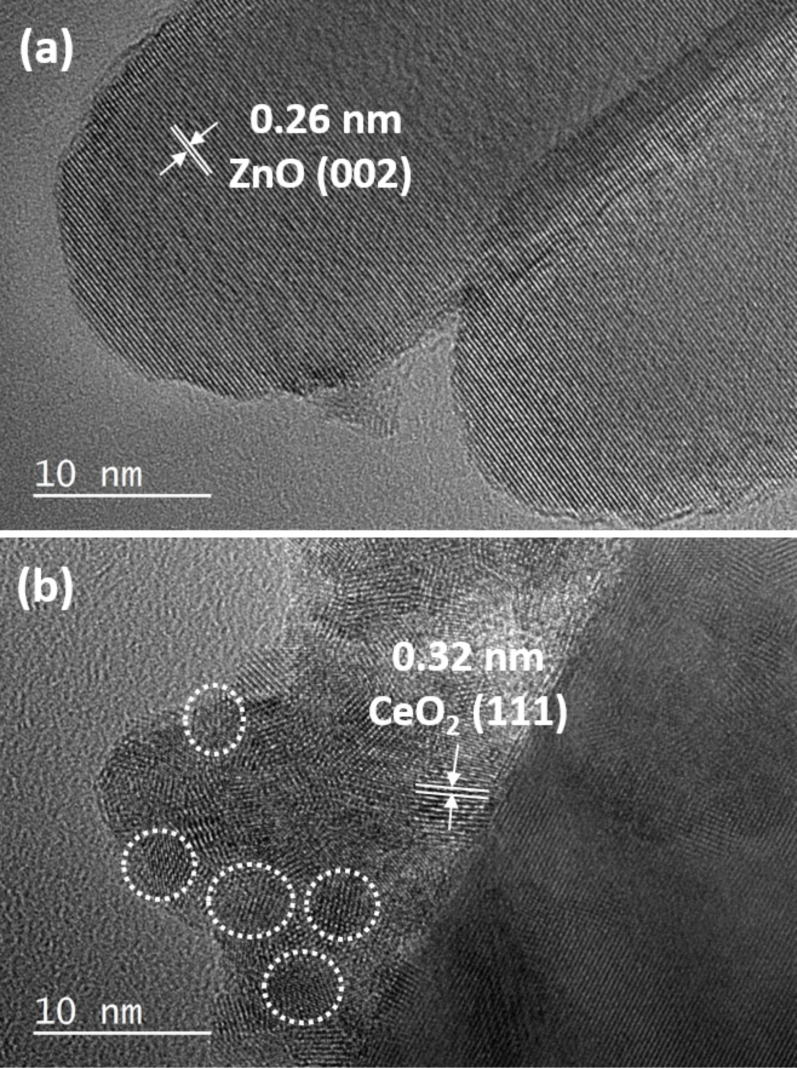
(a) HRTEM image of 5% Ce-doped ZnO rods, and (b) TEM image of the 10% Ce-doped ZnO rods showing CeO_2_ nanoparticles at the periphery of the rods.

Scanning electron microscopy (SEM) images of ZnO and ZnO:Ce particles indicate that all materials are composed of 1D rod-like ZnO particles of good quality (Figure S2 in the Supporting Information File 1). This was further confirmed by transmission electron microscopy (TEM) experiments (Figure 5). ZnO rods have a smooth surface, an average length of 130 nm and a diameter of 23 nm (Figure 5a). Contrary to hydrothermal methods recently developed for the production of ZnO:Ce particles [32], the increase in Ce doping does not induce a change in morphology from rods to spheres. An increase in rods length was observed when increasing the Ce doping (ca. 175 nm for the 10% doping) and some irregular cylindrical structures developed. The high crystallinity of the particles is further evident from the HRTEM image (Figure 6) and from the selected area electron diffraction (SAED) patterns shown in the insets of Figure 5. The bright and clear diffraction spots belong to the single crystal ZnO rods. From the HRTEM image (Figure 6a), one can clearly observe the crystal planes of ZnO. The interplanar spacing of ZnO is of ca. 0.26 nm, corresponding well to the (002) plane of ZnO. For 5, 7 and 10% doping in Ce, the ZnO rods were found to coexist with a significantly reduced population of small ellipsoidal CeO_2_ particles with an average diameter of ca. 5 nm deposited at the surface of the rods (Figure 6b), forming CeO_2_/ZnO:Ce heterostructures. The analysis of the interplanar distance calculated from the HRTEM image shows the (111) plane of cubic ceria.

**Figure 7:**
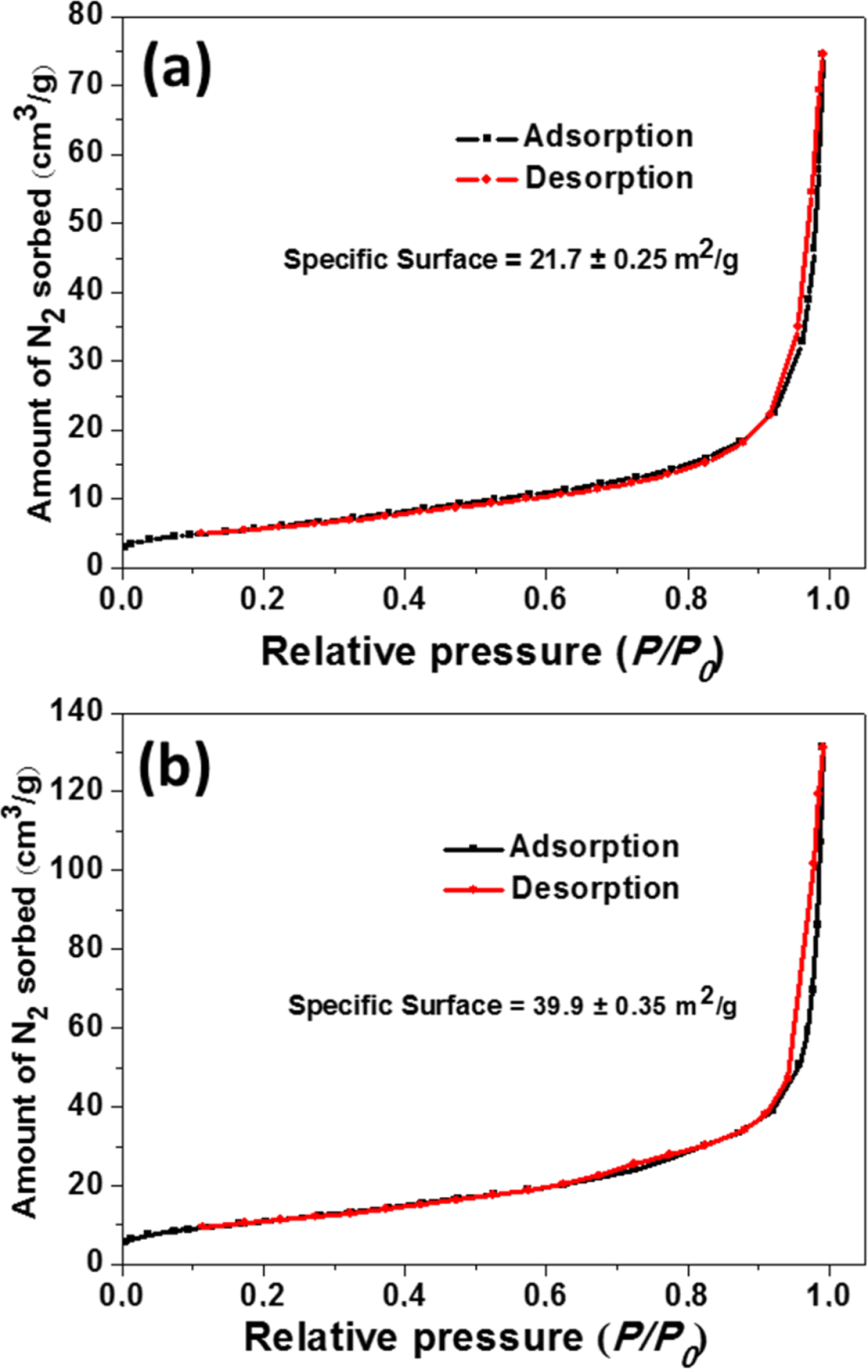
N_2_ adsorption/desorption curves at 77 K for ZnO and 5%-doped ZnO rods, giving surface areas of 21.7 and 39.9 m^2^/g, respectively. Black and red data correspond to the adsorption and desorption branches, respectively.

The BET surface area of pure ZnO and 5% doped ZnO:Ce rods were also investigated using nitrogen adsorption–desorption experiments (Figure 7). The N_2_ adsorption–desorption isotherms are of type II for both materials, according to the Brunauer–Dening–Dening–Teller (BDDT) classification [55]. Both materials exhibit high adsorption at relative pressures *P/P*_0_ close to 1.0, suggesting the formation of large mesopores and macropores. The 5% Ce-doped rods exhibit a higher surface area than ZnO rods (21.7 ± 0.2 m^2^/g and 39.9 ± 0.3 m^2^/g for ZnO and ZnO:Ce, respectively). The large specific surface of Ce:ZnO rods combined to the ability of the Ce dopant to reduce charge recombinations is promising for the efficient photodegradation of pollutants.

### Photocatalytic degradation of Orange II

**Figure 8:**
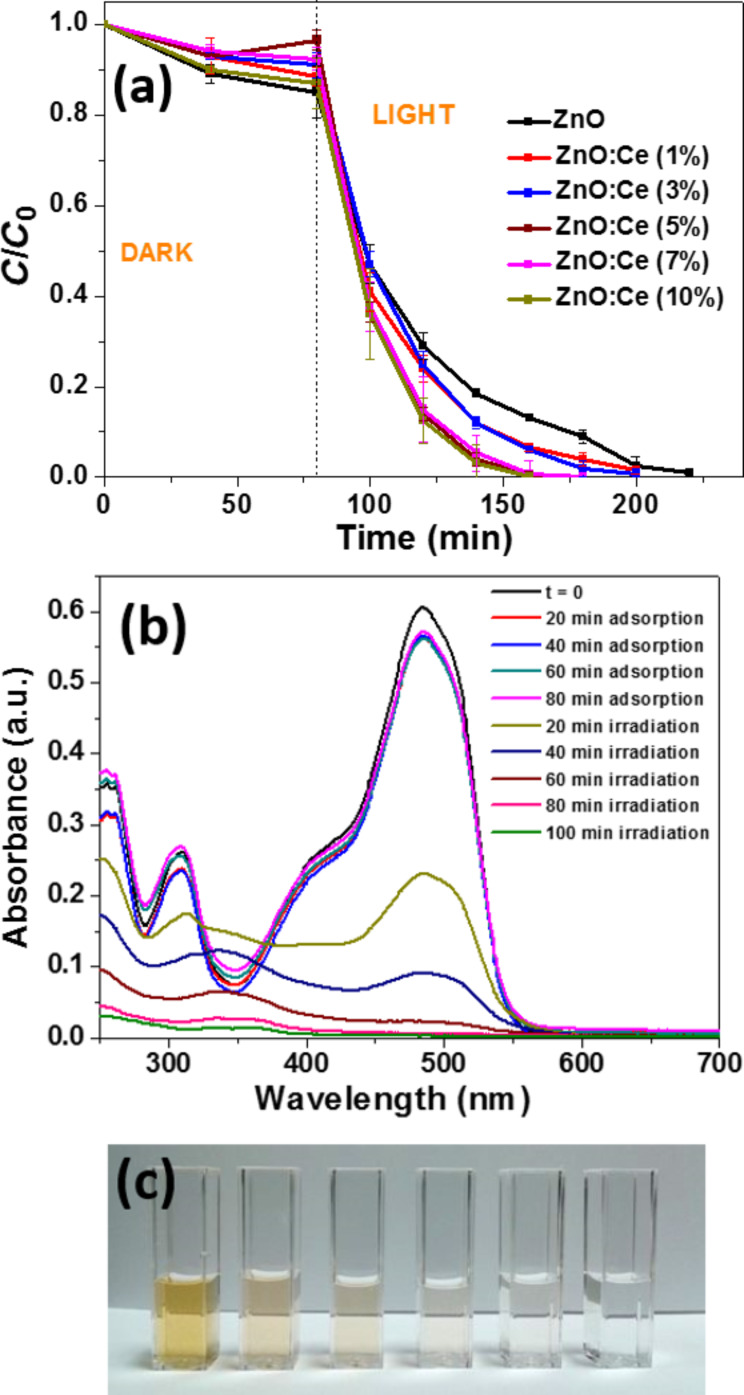
(a) Influence of the doping in Ce of ZnO rods for the degradation of Orange II in aqueous solution (*C* is the Orange II concentration at time *t*, and *C*_0_ is the concentration of the dye at *t* = 0; Volume of solution, 30 mL; Mass of photocatalyst, 30 mg; Orange II concentration, 10 mg/L). (b) Variation of Orange II concentration as a function of irradiation time. (c) Photographs of the Orange II solution during photocatalysis.

We first investigated the photocatalytic activities of Ce-doped ZnO in comparison to ZnO rods in the photodegradation of Orange II used at a 10 mg/L concentration. Initial control experiments showed that (i) solar light irradiation (5 mW/cm^2^) in the absence of any photocatalyst does not bleach Orange II and (ii) that the concentration of Orange II remained quite unchanged in the presence of the photocatalyst without light irradiation. As can be seen from Figure 8, under light irradiation, 5, 7 and 10% Ce-doped ZnO rods exhibit the highest photocatalytic activity and the degradation is nearly complete after 80 min irradiation. Figure 8b shows the decline of the characteristic absorption band of Orange II located at 485 nm using the ZnO:Ce (5%) catalyst and demonstrates that the photodegradation is complete (see also Figure 8c). The ln(*C*/*C*_0_) plots show a linear relationship with the irradiation time, indicating that the photodegradation of Orange II occurs via a pseudo-first-order kinetic reaction ln(*C*/*C*_0_) = −*kt*, where *k* is the photodegradation rate constant (min^−1^) and *C*_0_ and *C* are the concentrations of Orange II at time 0 and *t*, respectively. The rate constants *k* determined for the bleaching of 30 mL of a 10 mg/L dye solution were found to be 0.029, 0.032, 0.039, 0.063, 0.043 and 0.055 min^−1^ for ZnO and ZnO:Ce rods doped with 1, 3, 5, 7, and 10% Ce, respectively (see Figure S3 in the Supporting Information File 1 for the plots of ln(*C*_0_/*C*) vs reaction time). Based on these results, ZnO rods doped with 5% Ce were used in further experiments.

Because Ce:ZnO rods exhibit an extended photoresponding range in the visible region compared to ZnO (Figure 4), we also evaluated the photodegradation of Orange II under visible light (intensity = 5 mW/cm^2^) (Figure S4 in the Supporting Information File 1). Results obtained demonstrate that 78% of the dye was decomposed after 400 min and that the photodegradation required a longer time that under solar light irradiation.

### Effect of pH of the Orange II solution

**Figure 9:**
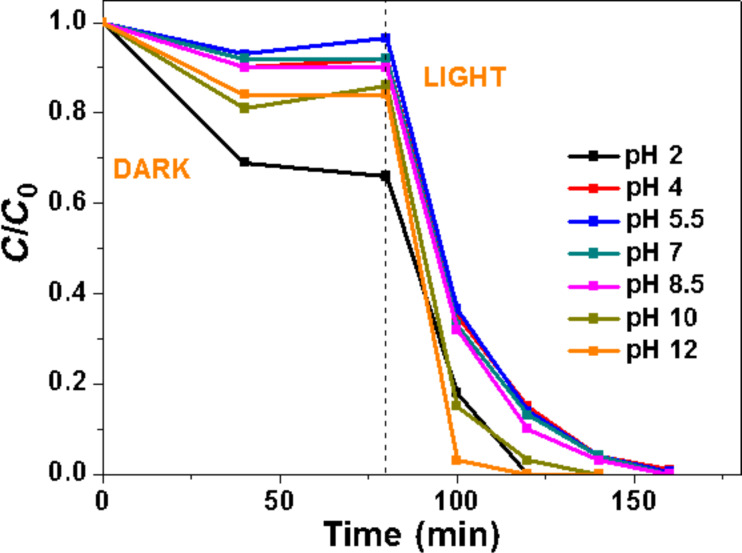
Influence of the pH of the Orange II solution on the photocatalytic activity of 5% Ce-doped ZnO rods.

The influence of the initial pH value of the Orange II solution on the photocatalytic activity was next studied (all other parameters were kept constant) (Figure 9). The pH value of the solution was adjusted before the adsorption phase and was not controlled during the reaction course. At pH values ranging from 4 to 8.5, no significant differences were observed in the photocatalytic activity. The adsorption of Orange II is increased at pH 2 and the dye bleached in 40 min. ZnO is known to be of modest stability in acidic medium, slowly dissolves and thus exhibits decreased catalytic activity [56]. The result obtained at pH 2 shows that Ce-doped ZnO rods exhibit higher stability at low pH than ZnO. The adsorption of the dye was also slightly increased at pH 10 and 12 and Orange II was decomposed in short times (60 and 40 min at pH 10 and 12, respectively). The high photocatalytic activity of the Ce-doped ZnO rods at basic pH may be attributed to the increased concentration of hydroxy anions that facilitate the photogeneration of hydroxy ^•^OH radicals (^−^OH + h^+^ → ^•^OH), thus enhancing the photocatalytic degradation efficiency.

### Effects of Orange II and of the photocatalyst concentrations

**Figure 10:**
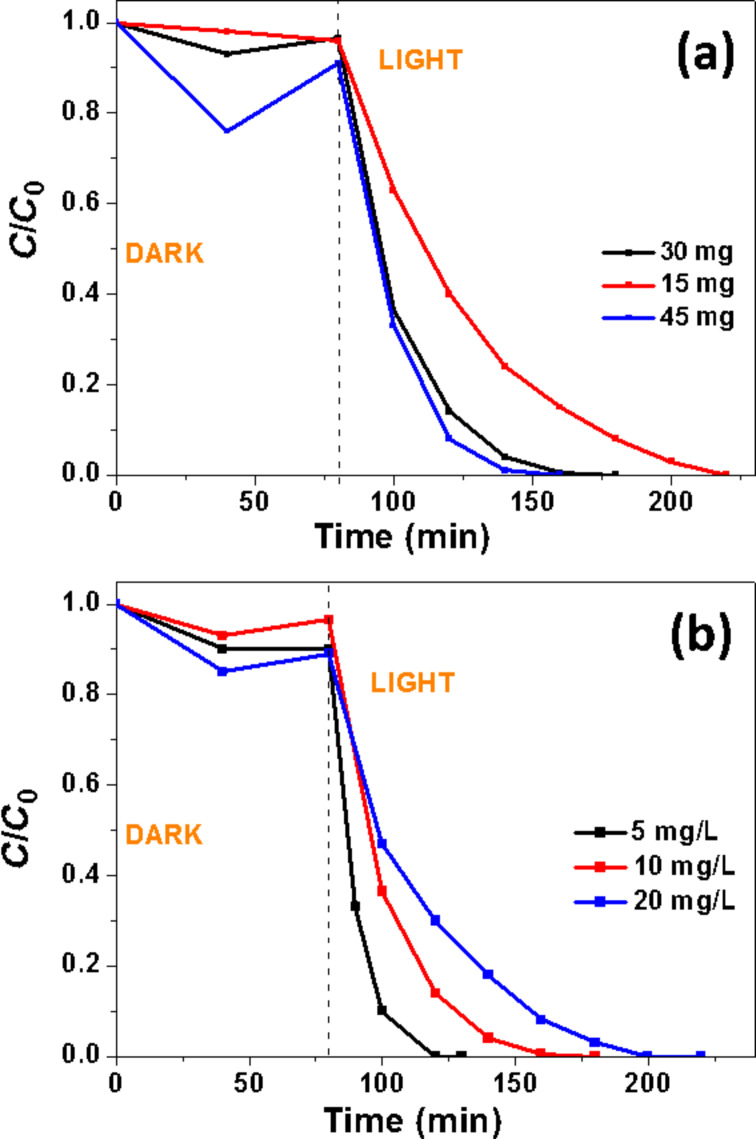
Effects of (a) the catalyst amount and (b) the dye concentration on the photocatalytic activity of 5% Ce-doped ZnO rods.

The effect of the mass of photocatalyst used for the degradation of Orange II was first evaluated (Figure 10a). Results obtained show that similar decomposition rates were obtained when using 30 or 45 mg of the catalyst (*k* = 0.063 and 0.075 min^−1^, respectively) while the efficiency of the photodegradation decreases when using only 15 mg of the catalyst (*k* = 0.027 min^−1^) (see Figure S5 in the Supporting Information File 1 for the plots of ln(*C*_0_/*C*) vs reaction time). The effect of the initial Orange II concentration (5, 10 or 20 mg/L) on the photodegradation under solar light irradiation was next investigated (Figure 10b). The decomposition rate of the dye was found to decrease with the increase of the dye concentration (*k* = 0.1, 0.063 and 0.032 min^−1^ for Orange II concentrations of 5, 10 and 20 mg/L, respectively) (see Figure S6 in the Supporting Information File 1 for the plots of ln(*C*_0_/*C*) vs reaction time). This decrease of the catalytic activity when increasing the initial Orange II concentration results from an increased adsorption of the dye on the catalyst surface. In addition, the incident photons may also be absorbed by the Orange II molecules in solution (filter effect), thus decreasing the amount of light available for the production of reactive oxygen species (ROS) at the surface of the photocatalyst.

### Influence of salts and molecules on the photocatalytic efficiency

**Figure 11:**
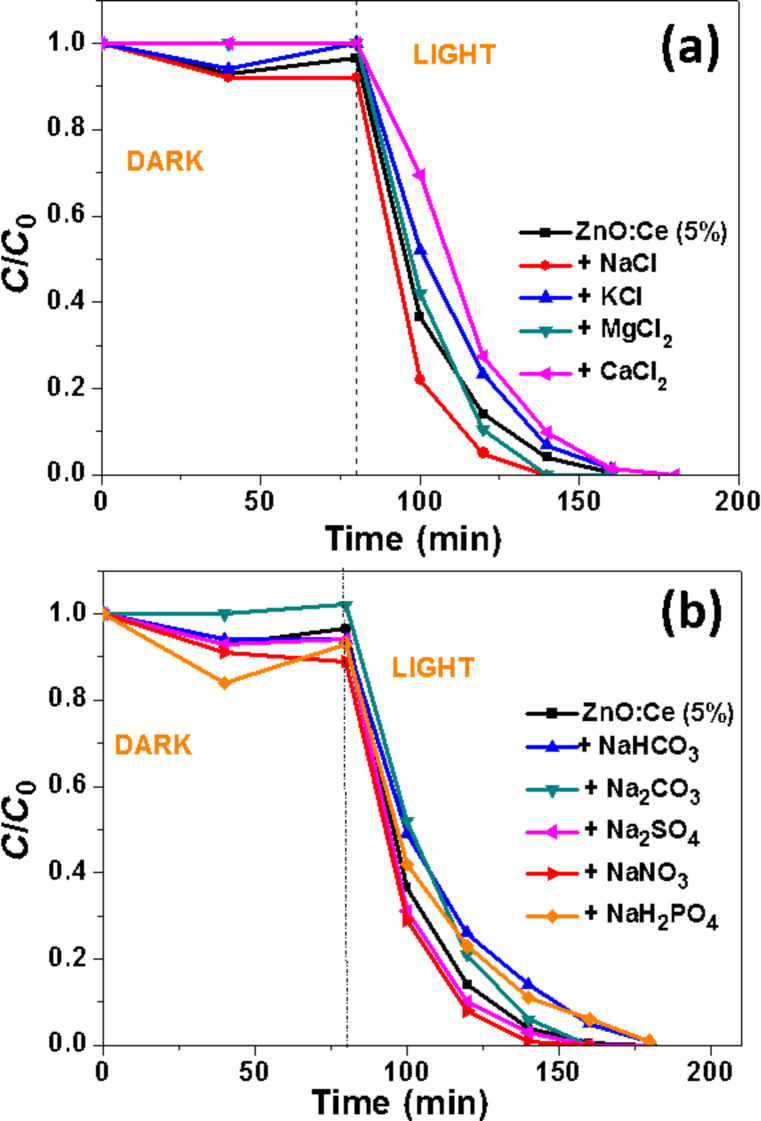
Effects of various salts on the photocatalytic efficiency of the ZnO:Ce rods used under solar light irradiation (Volume of solution, 30 mL; Orange II concentration, 10 mg/L; mass of catalyst, 30 mg).

The performance of Ce:ZnO rods for the photodegradation of Orange II in the presence of interfering substances like salts present in wastewater was also investigated. Numerous studies demonstrated that salts do not also adsorb on the photocatalyst surface but may also trap ROS and thus affect the photodegradation rate [57]. In a first set of experiments, we investigated the influence of various chlorides (NaCl, KCl, MgCl_2_ and CaCl_2_) used at a 10 mM concentration and at neutral pH on the photocatalytic activity of ZnO:Ce (5%) rods (Figure 11a). The amount of dye adsorbed by the photocatalyst and the photocatalytic activity are only slightly influenced by CaCl_2_ (complete degradation in 100 min while in the absence of CaCl_2_, the oxidation is complete in 80 min). Noteworthy is that NaCl and MgCl_2_ increased the photodegradation rate. We assume that Na^+^ or Mg^2+^ ions can either neutralize the negative sites at the surface of ZnO:Ce and thus diminish the electrostatic repulsion of Orange II with the catalyst or that these cations increase the amount of dye at the surface of the catalyst due to electrostatic interactions between the negatively charged Ce-doped ZnO, the cation and the anionic Orange II dye. We also varied the nature of the anions (S_2_^−^, HCO_3_^−^, CO_3_^2−^, SO_4_^2−^, NO_3_^−^ and H_2_PO_4_^−^) keeping Na^+^ as cation and maintaining the pH value at 7.0 (Figure 11b). As previously, the influence of these salts on the photocatalytic efficiency was found to be modest and no strong inhibition was observed. These results demonstrate that the ZnO:Ce photocatalyst is only weakly sensitive to salts commonly present in wastewater. Noteworthy is also that the Cl^−^ and SO_4_^2−^ anions, which are well-known to be ^•^OH radicals scavengers, have no detrimental effect on the photocatalytic activity.

**Figure 12:**
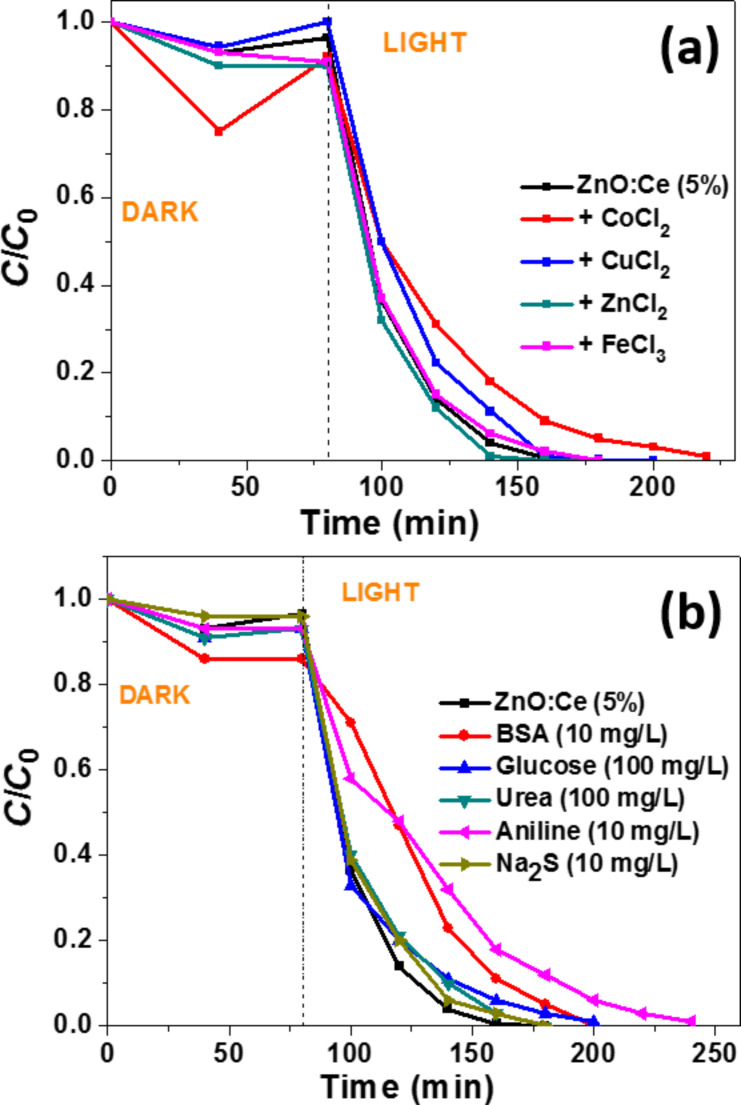
Effects of (a) transition metal salts and (b) of organic compounds and Na_2_S on the photocatalytic efficiency of the ZnO:Ce rods used under solar light irradiation (Volume of solution, 30 mL; Orange II concentration, 10 mg/L; mass of catalyst, 30 mg).

Transition metal salts used at 100 µM concentration like ZnCl_2_ or FeCl_3_ had no influence on the photocatalytic kinetic while CuCl_2_ and CoCl_2_ hindered the degradation process probably due (i) to their ability to consume photogenerated e^−^ and (ii) because the reduced cations obtained can trap holes and thus decrease the production of ^•^OH radicals and the reaction rates (Figure 12a) [58]. Glucose, a reducing sugar (100 mg/L), urea (100 mg/L) or Na_2_S (10 mg/L) only slightly decreased the photodegradation rate of Orange II at pH 7 (Figure 12b). Bovine serum albumin (BSA) (10 mg/L) and aniline (10 mg/L) had a more pronounced inhibition effect (degradation of Orange II in 120 and 160 min, respectively).

### Photocatalyst reusability

**Figure 13:**
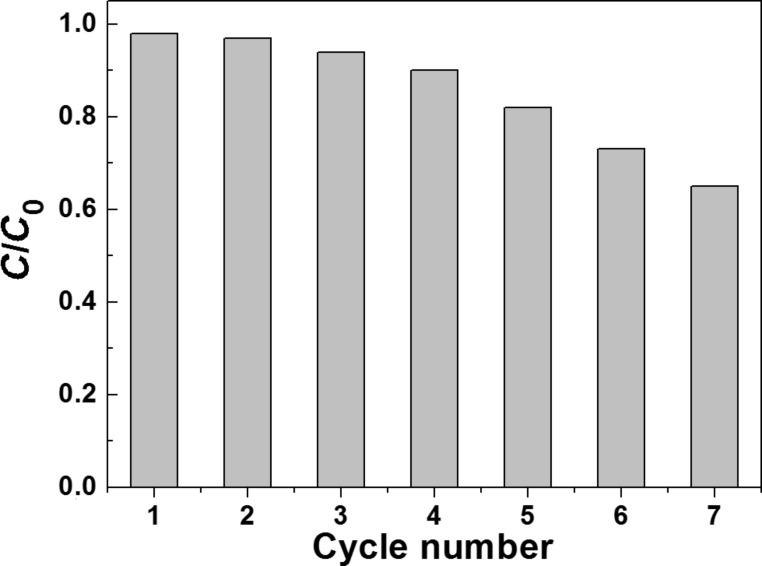
Recyclability of the ZnO:Ce photocatalyst.

The recycling behavior of ZnO:Ce rods has also been studied. For this purpose, seven successive photocatalytic experiments were conducted using 30 mg of the same catalyst and by changing the Orange II solution after each cycle. After 1 h irradiation, the reaction mixture was centrifuged and the UV–visible absorbance of Orange II at 485 nm measured. The catalyst recovered by centrifugation was reused after a simple washing with water. As can be seen in Figure 13, the photocatalytic activity is retained over 85% of its original value after five successive runs, which indicates the good stability of ZnO:Ce rods. Only after seven cycles, *C*/*C*_0_ decreased to ca. 0.65.

### Photocatalytic degradation mechanism

**Figure 14:**
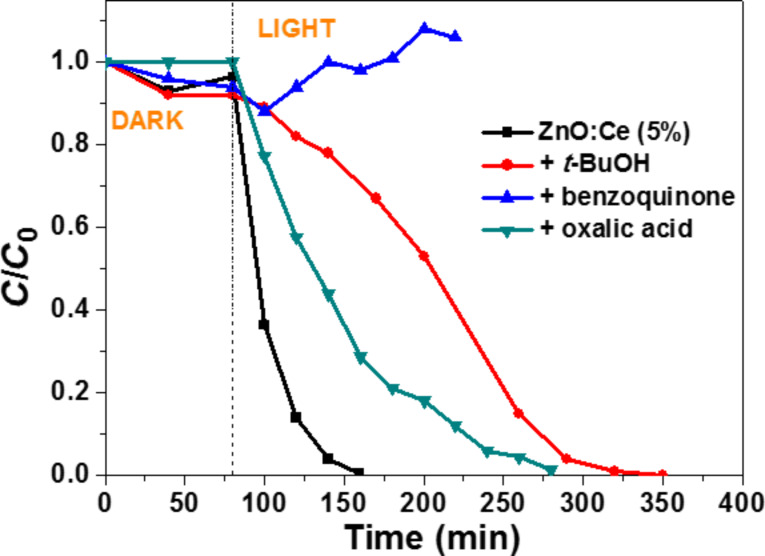
Influence of *t*-BuOH, benzoquinone and oxalic acid used as ^•^OH, O_2_^•−^ and h^+^ scavengers, respectively, on the photocalytic activity of the ZnO/Ce rods.

Scavenging experiments of the active species (^•^OH and O_2_^•−^ radicals, e^−^ and h^+^) were conducted to establish the mechanism of the photocatalytic degradation. When *t*-BuOH, a ^•^OH radical scavenger [59] was added at a 40 mL/L concentration, the kinetic of photocatalytic degradation of Orange II was reduced and required ca. 3.4-fold more time compared to the experiment conducted in the absence of *t*-BuOH (Figure 14). The strong inhibition of the photodegradation in the presence of *p*-benzoquinone (used at a 2.5 g/L concentration) [60], indicates that O_2_^•−^ radicals (or the species derived like hydroperoxide HO_2_^•^ or H_2_O_2_ obtained after reaction with H^+^) are the major active species in photocatalysis mediated by ZnO:Ce rods. Note that there is an overlap at 485 nm between the UV–visible absorption of Orange II and the oligohydroquinones originating from the O_2_^•−^-mediated polymerization of *p*-benzoquinone. Finally, the addition of oxalic acid used as h^+^ scavenger [61] (even used at the high concentration of 20 g/L) influenced less the degradation efficiency than *p*-benzoquinone or *t*-BuOH, indicating that direct oxidation of Orange II by reaction with h^+^ (Orange II + h^+^ → Orange II^+^) has only a modest role in the degradation pathway of the dye.

**Scheme 2:**
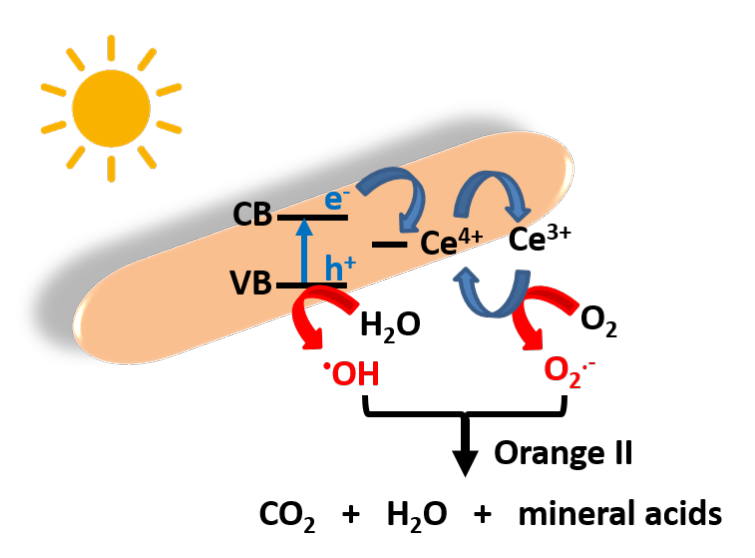
Schematic representation of the photocatalytic activity of ZnO:Ce rods.

On the basis of previously described results, the photocatalytic mechanism described in Scheme 2 can be proposed. Under solar or visible light excitation, e^−^ are transferred from the VB to the CB of ZnO, leaving h^+^ in the VB. Generally, only a weak part of these charge carriers migrates from the core to the surface of particles and reacts with O_2_ and H_2_O. Due to the Ce doping of ZnO rods, the photo-generated e^−^ can transfer to 4f energetic levels of Ce^4+^ acting as electron trap, thus decreasing photoinduced e^−^/h^+^ recombination. Ce^3+^ produced after reduction of Ce^4+^ or Ce^3+^ ions present in the rods after the solvothermal synthesis can react with dissolved O_2_ to generate superoxide anions and thus regenerate Ce^4+^. In the meantime, the h^+^ in the VB of ZnO react with H_2_O to produce hydroxyl radicals or with Orange II (direct oxidation pathway). The O_2_^•−^, ^•^OH and h^+^ species oxidize Orange II into CO_2_, water and mineral acids. Noteworthy is also the presence of mesopores and macropores in the photocatalyst and its relatively high BET surface area which help not only to concentrate the Orange II molecules at the surface of the catalyst but also scatter light between ZnO:Ce rods.

## Conclusion

A simple, efficient and cost-effective method to produce Ce-doped ZnO rods has been developed using a solvothermal method. Ce-doping not only increases the surface area of photocatalysts but also induces a red-shift in the absorption and improves solar and visible light capacities. At the optimal Ce doping percentage of 5 mol %, Orange II degradation is complete in 80 min. under solar light irradiation and the ZnO:Ce rods exhibit much higher photocatalytic activity than pure ZnO rods. This high photocatalytic efficiency is associated to the decrease of electron/hole recombination, to the small size of ZnO:Ce rods and to their high specific surface compared to ZnO particles. Furthermore, ZnO:Ce rods exhibit good stability and can reused at least seven times, thus indicating that these materials have great potential as photocatalysts in practical applications.

## Experimental

### Materials

Zn(OAc)_2_·2H_2_O (>98%, Sigma), anhydrous Ce_2_(SO_4_)_3_ (97%, Sigma), Orange II sodium salt (>85%, Sigma), sodium hydroxide (>97%, Sigma), *tert*-butanol (*t*-BuOH, 99%, Sigma), *p*-benzoquinone (>98%, Sigma), oxalic acid (>99%, Sigma) and anhydrous ethanol were used as received without further purification. All solutions were prepared using Milli-Q water (18.2 MΩ·cm, Millipore) as solvent.

### Preparation of ZnO and ZnO:Ce nanorods

In a similar manner as described before [7,13], ZnO rods were synthesized by a solvothermal method based on the hydrolysis of Zn(OAc)_2_. Typically, in a three-necked flask equipped with a condenser and a dropping funnel, Zn(OAc)_2_·2H_2_O (511 mg, 2.33 mmol) was dissolved in 35 mL ethanol. To this solution, NaOH (466 mg, 11.65 mmol) in 35 mL ethanol was added dropwise and the mixture was stirred for 30 min at room temperature. Then, the mixed solution was transferred into a 140 mL Teflon-sealed autoclave and was heated at 160 °C in an electrical oven for 24 h. After allowing to cool naturally, the ZnO rods were collected by centrifugation, washed three times with water, one time with ethanol, and dried at 70 °C overnight. Typically, this procedure affords 150 mg of ZnO rods.

Ce-doped ZnO rods were prepared using a similar synthetic procedure. For the rods doped with 5% Ce, Zn(OAc)_2_·2H_2_O (485 mg, 2.215 mmol) and Ce_2_(SO_4_)_3_ (66.22 mg, 0.116 mmol) were used. The purification and drying procedures are similar to those previously described for ZnO rods.

### Photocatalytic degradation of Orange II

In a similar manner as described before [7,13], the photocatalytic activity was evaluated by the degradation of an aqueous Orange II solution (10 mg/L) at room temperature under solar light irradiation. In a typical experiment, the ZnO:Ce rods (30 mg) were dispersed in 30 mL Orange II aqueous solution and the suspension was magnetically stirred under ambient conditions for 80 min in the dark to reach an adsorption-desorption equilibrium. Under stirring, the suspension was exposed to simulated solar light irradiation produced by Sylvania LuxLine FHO T5 neon tubes. The light intensity was controlled using a radiometer (the distance between the lamps and the captors or the surface of the Orange II solution was controlled in each experiment to fix the light intensity at 5 mW/cm^2^). At various irradiation periods, 1 mL of the solution was extracted and centrifuged (15000 rpm for 2 min) to remove the photocatalyst. The degradation process was monitored by measuring the UV–visible absorption of Orange II at 485 nm. For experiments conducted under visible light irradiation, a polycarbonate filter was used to cut off any light radiation below the wavelength of 400 nm.

### Characterization

TEM images were taken by placing a drop of the particles dispersed in water onto a carbon film-supported copper grid. Samples were studied using a Philips CM200 instrument operating at 200 kV. SEM pictures were prepared using JEOL Scanning Electron Microscope JSM-6490 LV. The XRD data were collected from an X'Pert MPD diffractometer (Panalytical AXS) with a goniometer radius 240 mm, fixed divergence slit module (1/2° divergence slit, 0.04 rd Sollers slits) and an X'Celerator as a detector. The powder samples were placed on a silicon zero-background sample holder and the XRD patterns were recorded at room temperature using Cu Kα radiation (λ = 0.15418 nm). XPS analyses were performed on a Gammadata Scienta (Uppsala, Sweden) SES 200-2 spectrometer under ultra-high vacuum (*P* < 10^−9^ mbar). The measurements were performed at normal incidence (the sample plane is perpendicular to the emission angle). The spectrometer resolution at the Fermi level is about 0.4 eV. The depth analyzed extends up to about 8 nm. The monochromatized Al Kα source (1486.6 eV) was operated at a power of 420 W (30 mA and 14 kV) and the spectra were acquired at a take-off angle of 90° (angle between the sample surface and photoemission direction). During acquisition, the pass energy was set to 500 eV for wide scans and to 100 eV for high-resolution spectra. CASAXPS software (Casa Software Ltd, Teignmouth, UK, http://www.casaxps.com) was used for all peak fitting procedures and the areas of each component were modified according to classical Scofield sensitivity factors.

The textural properties of the materials were investigated with a Micromeritics 3Flex Surface Characterization Analyzer instrument using liquid nitrogen (−196 °C). Prior to the analyses, the samples were out-gassed overnight under primary vacuum at 40 °C on the ports of the Micromeritics VacPrep 061 degasser followed by 4 h out-gassing under high vacuum on the analyse ports. The resulting isotherms were analysed using the BET (Brunauer–Emmett–Teller) method.

All the optical measurements were performed at room temperature (20 ± 1°C) under ambient conditions. Absorption spectra of liquid samples were recorded on a Thermo Scientific Evolution 220 UV–visible spectrophotometer. DRS were recorded on a Shimadzu 2600 UV–visible spectrophotometer. BaSO_4_ powder was used as a standard for baseline measurements and spectra were recorded in the range of 250–1400 nm. Raman spectra were recorded using a Xplora spectrometer from Horiba Scientific with 532 nm wavelength incident YAG laser light.

## Supporting Information

File 1 Additional experimental data.
