# Supplementary material for: High performance Ce-doped ZnO nanorods for sunlight-driven photocatalysis
Source: Beilstein J Nanotechnol. 2016 Sep 26;7:1338–49. doi: 10.3762/bjnano.7.125 (PMC5082445; doi:10.3762/bjnano.7.125)
Supplement: File 1 — Additional experimental data. [file Beilstein_J_Nanotechnol-07-1338-s001.pdf]

Supporting Information

for

High performance Ce-doped ZnO nanorods for sunlight-driven photocatalysis

Bilel Chouchene[1], Tahar Ben Chaabane[1], Lavinia Balan[2], Emilien Girot[3], Kevin Mozet[3], Ghouti Medjahdi[4] and Raphaël Schneider*[3]

Address: [1]Unité de Recherche Synthèse et Structure de Nanomatériaux UR 11 ES 30, Université de Carthage, Faculté des Sciences de Bizerte, 7021 Jarzouna, Bizerte, Tunisia, [2]Institut de Science des Matériaux de Mulhouse (IS2M), CNRS, UMR 7361, 15 rue Jean Starcky, 68093 Mulhouse, France, [3]Université de Lorraine, Laboratoire Réactions et Génie des Procédés (LRGP), UMR 7274, CNRS, 1 rue Grandville, BP 20451, 54001 Nancy Cedex, France and [4]Institut Jean Lamour (IJL), Université de Lorraine, CNRS, UMR 7198, CNRS, BP 70239, 54506 Vandoeuvre-lès-Nancy Cedex, France

Email: Raphaël Schneider - raphael.schneider@univ-lorraine.fr

* Corresponding author

Additional experimental data

Table S1: Lattice parameters of ZnO and ZnO:Ce rods.

Doping in Ce (%)	a (Å)	c (Å)	Lattice volume (Å^3)
0	3.244	5.197	47.35
1	3.246	5.201	47.44
3	3.253	5.212	47.78
5	3.254	5.213	47.80
7	3.250	5.206	47.61
10	3.249	5.206	47.61

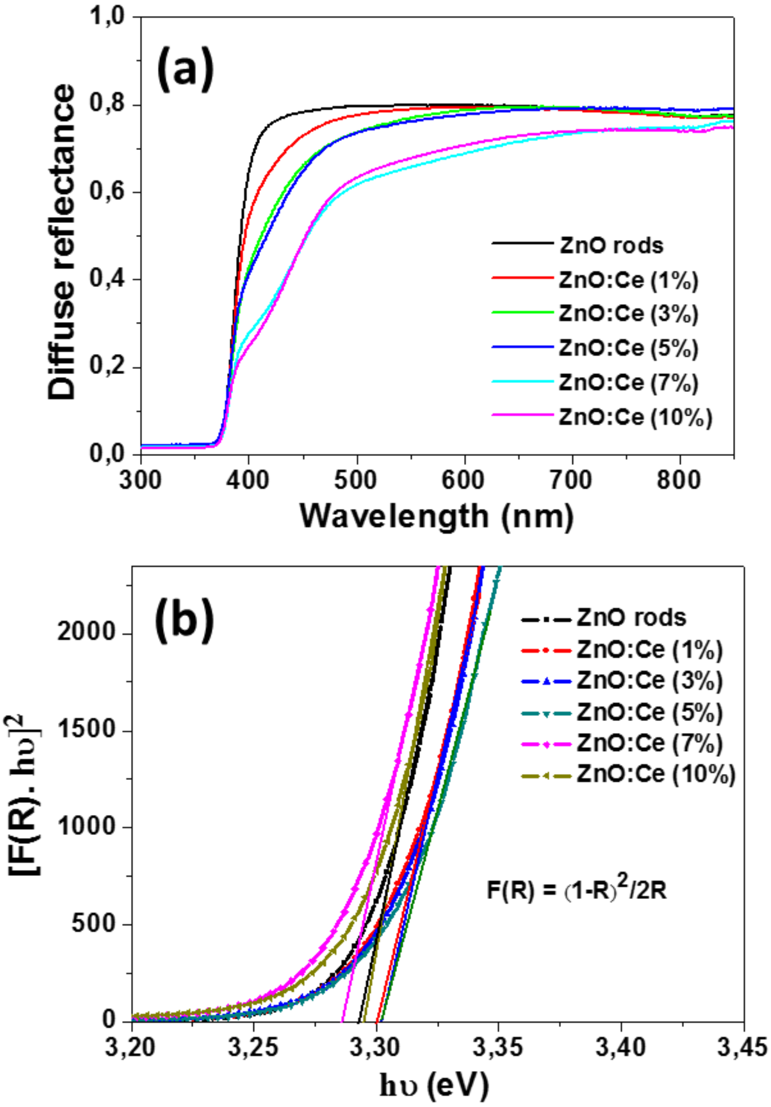

Figure S1: (a) UV–visible absorption spectra of ZnO and ZnO:Ce rods, (b) Tauc plots of ZnO and ZnO:Ce rods for the determination of bandgap energy values.

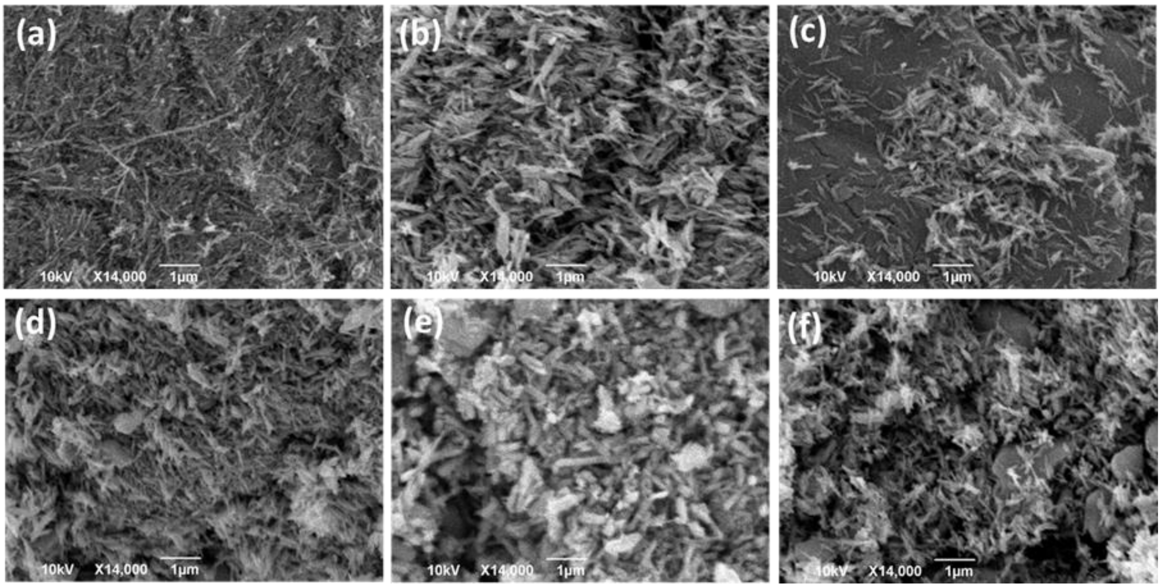

Figure S2: SEM images of (a) ZnO rods and (b–f) ZnO rods doped with 1, 3, 5, 7 and 10% Ce, respectively.

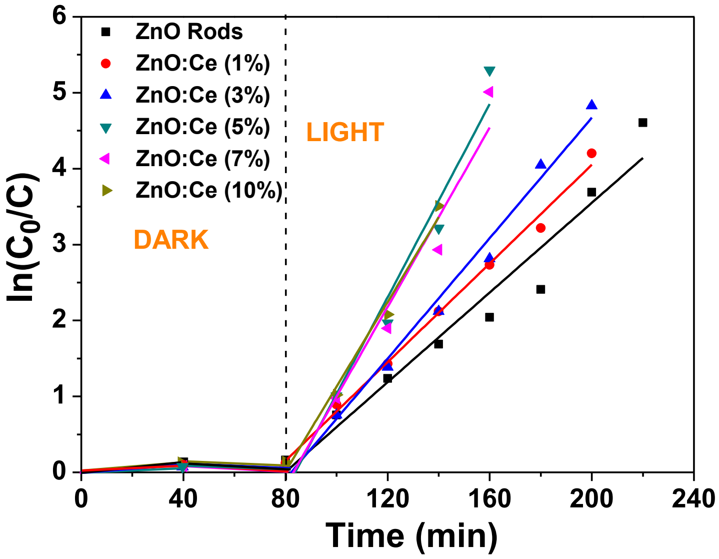

Figure S3: Plots of ln(C_0/C) of Orange II versus reaction time when varying the dopant percentage in Ce in the photocatalyst (concentration of Orange II = 10 mg/L).

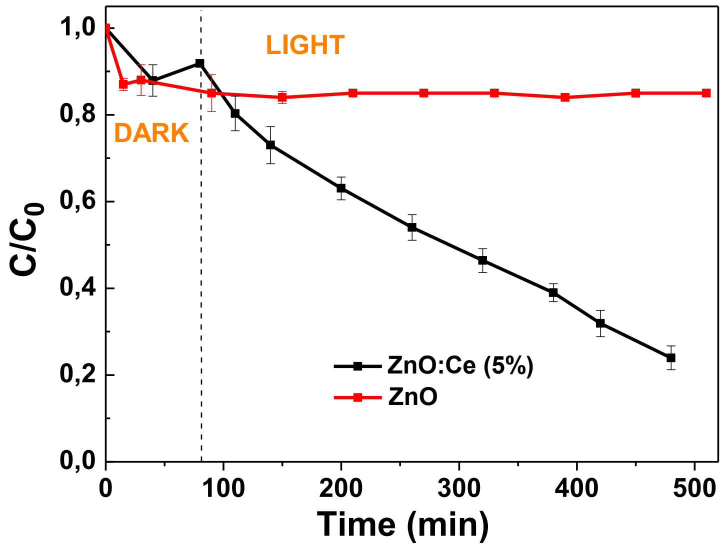

Figure S4: Comparison of the photocatalytic efficiencies under visible light irradiation of ZnO and 5% Ce-doped ZnO rods.

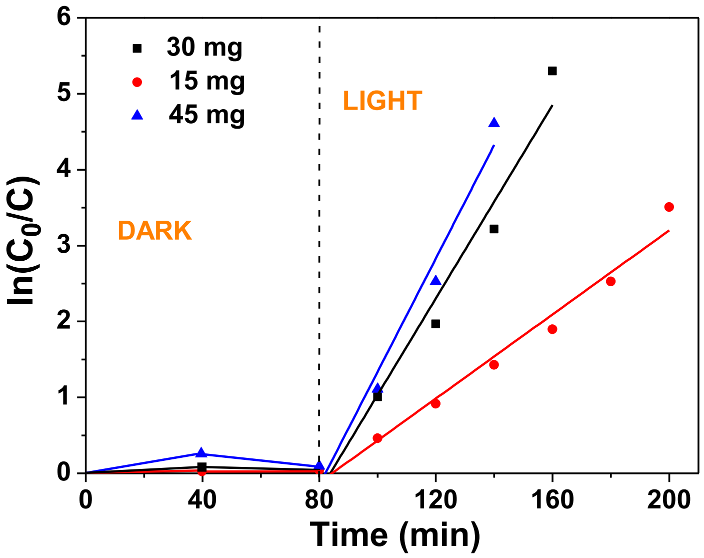

Figure S5: Plots of ln(C_0/C) of Orange II versus reaction time when varying the amount of ZnO:Ce catalyst (concentration of Orange II = 10 mg/L).

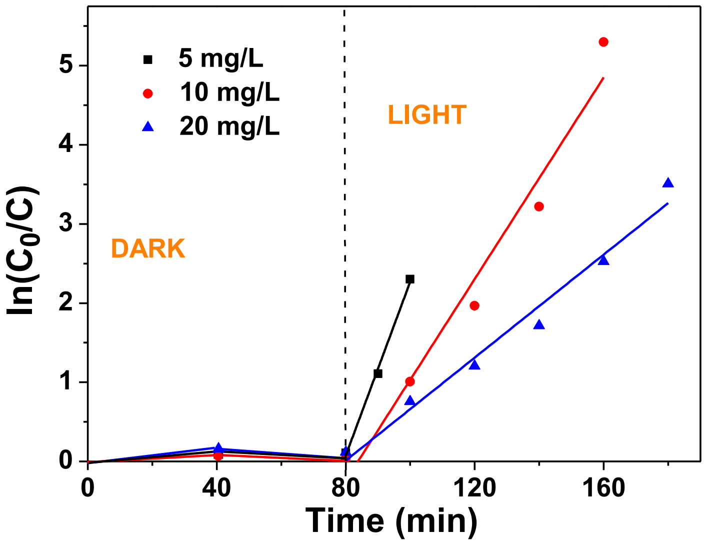

Figure S6: Plots of ln(C_0/C) of Orange II versus reaction time when varying the concentration of Orange II (5, 10 or 20 mg/L).
